# Expression of miRNAs in Pre-Schoolers with Autism Spectrum Disorders Compared with Typically Developing Peers and Its Effects after Probiotic Supplementation

**DOI:** 10.3390/jcm12227162

**Published:** 2023-11-18

**Authors:** Letizia Guiducci, Manuela Cabiati, Elisa Santocchi, Margherita Prosperi, Maria Aurora Morales, Filippo Muratori, Emioli Randazzo, Giovanni Federico, Sara Calderoni, Silvia Del Ry

**Affiliations:** 1 CNR, Institute of Clinical Physiology, 56124 Pisa, Italy; letizia.guiducci@cnr.it (L.G.); manuela.cabiati@cnr.it (M.C.); morales@ifc.cnr.it (M.A.M.); silvia.delry@cnr.it (S.D.R.); 2 UFSMIA Zona Valle del Serchio, Azienda USL Toscana Nord Ovest, 55032 Castelnuovo di Garfagnana, Italy; santocchielisa@gmail.com; 3 UFSMIA Valdera-Alta Val di Cecina, Azienda USL Toscana Nord Ovest, 56128 Pisa, Italy; margherita.prosperi@uslnordovest.toscana.it; 4 Department of Developmental Neuroscience, IRCCS Stella Maris Foundation, 56128 Pisa, Italy; filippo.muratori@fsm.unipi.it; 5 Unit of Pediatric Endocrinology and Diabetes, Department of Clinical and Experimental Medicine, University of Pisa, 56126 Pisa, Italy; emioli.randazzo@gmail.com (E.R.); giovanni.federico@med.unipi.it (G.F.); 6 Department of Clinical and Experimental Medicine, University of Pisa, 56126 Pisa, Italy

**Keywords:** microRNA, autism spectrum disorder, pre-schoolers, probiotics, real-time PCR

## Abstract

Alteration of the microbiota–gut–brain axis has been recently recognized as a possible contributor to the physiopathology of autism spectrum disorder (ASD). In this context, microRNA (miRNAs) dysfunction, implicated both in several neuropathological conditions including ASD and in different gastrointestinal disorders (GIDs), could represent an important modulating factor. In this contextual framework, we studied the transcriptional profile of specific circulating miRNAs associated with both ASD (miR-197-5p, miR-424-5p, miR-500a-5p, miR-664a-5p) and GID (miR-21-5p, miR-320a-5p, miR-31-5p, miR-223-5p) in a group of pre-schoolers with ASD and in typically developing (TD) peers. In the ASD group, we also assessed the same miRNAs after a 6-month supplementation with probiotics and their correlation with plasma levels of zonulin and lactoferrin. At baseline, the expression of miRNAs involved in ASD were significantly reduced in ASD pre-schoolers vs. TD controls. Regarding the miRNAs involved in GID, the expression levels of miR-320-5p, miR-31-5p, and miR-223-5p were significantly higher in ASD than in TD subjects, whereas miR-21-5p showed significantly reduced expression in the ASD group vs. TD group. Supplementation with probiotics did not significantly change the expression of miRNAs in the ASD population. We found a significative negative correlation between zonulin and miR-197-5p and miR-21-5p at baseline, as well as between lactoferrin and miR-223-5p after 6 months of probiotic supplementation. Our study confirms the presence of an altered profile of the miRNAs investigated in ASD versus TD peers that was not modified by supplementation with probiotics.

## 1. Introduction

Autism spectrum disorder (ASD) is a complex neurodevelopmental condition characterized by impaired social interaction and communication, as well as the presence of restricted interests, repetitive behavior, and alterations of sensory processing [1]. The worldwide median prevalence of ASD is approximately of 1% [2], with a strong male bias that is consistent across epidemiological studies [3]. In addition to core symptoms, ASD individuals are more likely to experience a number of comorbidities that can be psychiatric (including attention deficit hyperactivity disorder (ADHD), anxiety, and depressive disorders) or somatic (including epilepsy, gastrointestinal, and sleep disorders) [4]. These additional issues, along with further complicating clinical presentation and treatment, may negatively impact on quality of life and economic burden [5]. ASD diagnosis includes direct behavioral observation of the child and a detailed developmental history; no valid diagnostic biomarkers for ASD have been detected according to a recent systematic review encompassing biochemical, genetic, neuroimaging, neurophysiological, and neuropsychological measures [6].

The ASD etiopathogenesis is multifactorial and derives from a complex interplay of genetic susceptibility and pre-perinatal environmental factors [7,8]. In this context, the existing evidence supports the possible contributing role of epigenetic modifications, which have been detected in both syndromic and idiopathic ASD [9,10]. Specifically, epigenetic regulation coordinates gene expression (without inducing DNA sequence changes [11]) by influencing several genes [12], including those modulating neurogenesis and brain development [13]. Among the epigenetic regulators are microRNAs (miRNAs), which are small, noncoding RNAs regulating gene expression at the post-transcriptional level of various cellular processes, including those involved in neurodevelopment, by marking specific mRNAs to repress their translation or to induce their degradation [14]. They are abundantly present in the brain, and their dysfunction has been implicated in several neuropathological conditions including ASD. miRNAs, previously known to be expressed only in cells and tissues, have also been detected in extracellular body fluids such as serum, plasma, saliva, and urine [15]. Altered expression of cellular and circulating miRNAs has been observed in ASD individuals compared with typically developing (TD) controls, and they are now considered as potential targets for the development of ASD novel therapeutic strategies [15,16]. In particular, dysregulation of some miRNAs (miR-197-5p, miR-424-5p, miR-500a-5p, miR-664a-5p) is associated with ASD and these molecules can potentially constitute molecular biomarkers [17], whereas other miRNAs (miR-21-5p, miR-320a-5p, miR-31-5p, miR-223-5p) may serve as important causative factors of different gastrointestinal disorders [18].

In addition, the alteration of the microbiota–gut–brain axis has been recently recognized as a possible contributing factor in the etiopathogenesis of ASD [19,20,21,22,23,24,25,26]. Indeed, a considerable number of subjects with ASDs have significant gastrointestinal (GI) dysfunctions, including altered bowel habits and chronic abdominal pain (for a recent review, see Holingue et al., 2018) [27]. Previous studies have analyzed the associations between GI symptoms and ASD symptoms and had discrepant results: some investigations reported that children with and without GI problems did not differ in autism symptom severity [28,29,30], whereas others observed an association between GI symptoms and some altered behaviors [31,32,33,34,35]. An increased gut permeability, or “leaky gut”, might be implicated in favoring GI symptoms, allowing bacterial metabolites to cross the gut barrier and potentially impacting the early neurodevelopmental processes [36].

In this framework, some recent reviews have stressed the importance of taking probiotics in order to maintain gut homeostasis, to improve gut microbiota, and ultimately to confer several health benefits [37,38,39]. In the ASD field, probiotics have already been administered to children both in randomized controlled trials (RCT) and non-randomized interventions (for a recent systematic review, see [40]). Although the results of probiotic supplementation may not be translated into clinical recommendations, this intervention could be considered as an add-on option for patients with ASD [41] in association with evidence-based psychoeducational interventions [42].

Based on these assumptions, the current study aims to evaluate: (i) the transcriptional profile of specific circulating miRNAs in ASD pre-schoolers and in TD peers; (ii) whether the miRNA expression profile changed after 6-months of probiotic supplementation in ASD pre-schoolers divided according to the presence or absence of gastrointestinal symptoms; (iii) the concentrations of markers of intestinal barrier protection (circulating lactoferrin) and markers of increased intestinal permeability [43] in ASD children; and (iv) the possible relationships between miRNAs and the plasma levels of zonulin and lactoferrin.

## 2. Methods

### 2.1. Subjects and Plasma Collection

The study is part of a larger RCT on the effects of probiotic supplementation in ASD pre-schoolers [25,26]. Participants with ASD were recruited from the Child and Adolescence Mental Health Services of the Tuscany Region and the Unit of Child Psychiatry and the Unit of Child Rehabilitation of IRCCS Stella Maris Foundation (Pisa, Italy), a tertiary-care university hospital.

The inclusion criteria were age range between 18 and 72 months and ASD diagnosis performed by an experienced multidisciplinary team according to the criteria reported in the Diagnostic and Statistical Manual of Mental Disorders—5th Edition [1] (DSM-5). The exclusion criteria were neurological syndromes or focal neurological signs, history of birth asphyxia, severe premature birth or perinatal injuries, epilepsy, significant sensory impairment (e.g., blindness, deafness), diagnosis of not functional gastrointestinal disorder (e.g., gastroesophageal reflux, food allergies) or coeliac disease, special diets already underway (i.e., gluten-free diet, casein-free diet, high-protein diet, ketogenic diet), and known brain anomalies.

The study population was represented by 31 ASD pre-schoolers and by 10 sex/age-matched TD subjects who served as the control group. The age and the anthropometric characteristics are reported in Table 1.

ASD subjects were supplemented with probiotics for 6 months (De Simone Formulation (DSF)—Vivomixx^®^ in EU, Visbiome^®^ in USA—containing 450 billion bacteria of eight different probiotic strains: *Streptococcus thermophilus*, *Bifidobacterium breve*, *Bifidobacterium longum*, *Bifidobacterium infantis*, *Lactobacillus acidophilus*, *Lactobacillus plantarum*, *Lactobacillus paracasei*, *Lactobacillus delbrueckii subsp*. *Bulgaricus*).

ASD subjects were classified as belonging to the gastrointestinal group (GI) or to the non-GI (NGI) group on the basis of the Gastrointestinal Severity Index (GSI) [44]. Specifically, this questionnaire allows obtaining a composite score based on the severity of GI signs and symptoms (constipation, diarrhea, average stool consistency, stool smell, flatulence, abdominal pain, unexplained daytime irritability, night-time awakening, abdominal tenderness) reported by parents in the previous two weeks. We adopted a GSI cut-off of 4 (the range of this scale is 0–17), with at least 3 score points obtained from the first six items of the scale, evaluated by Adams et al. [45] as more specifically related to GI symptoms and named the 6-GI Severity Index (6-GSI).

In a previous study performed by our research group [15,26], we investigated possible changes in the gastrointestinal and ASD symptoms at the end of probiotic administration. Accordingly, the ASD population was subdivided in responders (R) and non-responders (NR) as regards to gastrointestinal (R-GI/NR-GI) and neurodevelopmental (R-ASD/NR-ASD) symptoms following an improvement (R) or an unchanged/worsening (NR) of the GI index and ADOS-calibrated severity score (ADOS-CSS) [46,47], respectively. In the present study, we examined the possible variations of both the circulating miRNA expression trend and lactoferrin and zonulin plasma levels in this cohort of ASD patients.

Typically developing children were healthy subjects referred as outpatients to the Unit of Pediatric Endocrinology and Diabetes, Department of Clinical and Experimental Medicine, University of Pisa, Italy, who repeated blood examinations after an intervening disease. At the time of blood sampling, they had not received drugs for at least one week and their bio-humoral parameters, including indices of inflammation, were in the normal range.

We collected blood samples from all the subjects by venipuncture performed the morning after overnight fasting. For ASD subjects, the samples were collected before (T0) and after 6 months of probiotic administration (T1) into an ethylene diaminetetraacetic acid (EDTA) (1 mg/mL) vacutainer. After collection, the samples were quickly separated by centrifugation for 15 min at 4 °C and the plasma was stored frozen at −80 °C in 1mL aliquots in polypropylene tubes until assay.

### 2.2. Zonulin and Lactoferrin Assays

Lactoferrin and Zonulin were measured in 10 μL and 100 μL of plasma, respectively, using immunometric assays (Zonulin ELISA kit, Elabscience and Lactoferrin ELISA (Human), DRG Diagnostics, Houston, TX, USA).

The assay sensitivities were 1.1 ng/mL and 0.47 ng/mL for lactoferrin and zonulin, respectively. Within-assay variability, evaluated for each kit, was <10% for both analytes (CK_Lattoferrin_: 717.7 ± 29.8 ng/mL (n = 4 duplicate assays, CV = 8.3%) and CK_Zonulin_: 50.5 ± 1.001 ng/mL (n = 4 duplicate assays, CV = 4%)). Assay accuracy was evaluated by dilution tests (1:5 and 1:2 serial dilutions for lactoferrin and for zonulin) and the linearity of the response was observed for both immunometric assays. Two control samples were assayed in each run for quality control.

### 2.3. miRNA Extraction, Reverse Transcription, and Real-Time PCR

Extraction of miRNAs was performed by using the miRNeasy Serum/Plasma Kit (Qiagen S.p.a., Milano, Italy). As previously reported [48], 200 μL of plasma was lysed in adequate lysis reagent and applied to silica-membrane columns that bind total RNA, allowing phenol and other contaminants to be efficiently washed away. To help to monitor RNA recovery and reverse transcription efficiency, a spike-in control was used as an internal control for plasma miRNA expression profiling. The miRNeasy Serum/Plasma spike-in control is a *C. elegans* miR-39 miRNA mimic that can be easily detected via real-time PCR using the miScript PCR System (Qiagen S.p.a., Milano, Italy) in combination with the Ce_miR-39_1 miScript Primer Assay (Qiagen S.p.a., Milano, Italy). High-quality RNA was then eluted in a small volume (14 μL) of RNase-free water; all miRNA samples were stored at −80 °C after evaluation of their integrity, purity, and concentration.

Mature miRNA sequences, used as forward primers for the detection of miRNAs, were downloaded from the miRBase database (v22.1, 2018-10) (www.mirbase.org) (Table 2).

Real-time PCR reactions were performed in duplicate using a Bio-Rad C1000^™^ thermal cycler system (CFX-96 Real-Time detection system, Bio-Rad Laboratories Inc., Hercules, CA, USA). In order to monitor cDNA amplification, a fluorogenic DNA binding dye was used (SsoFAST EvaGreen Supermix Bio-Rad Laboratories Inc., Hercules, CA, USA) and the optimal real-time PCR conditions were set for each miRNA analysed, except for Ce_miR-39_1, which was a standardized assay. To assess product specificity, amplicons were systematically checked by melting curve analysis (from 65 °C to 95 °C with increments of 0.5 °C/cycle). All experiments followed the MIQE (Minimum Information for Publication of Quantitative Real-Time PCR Experiments) guidelines [49].

## 3. Statistics

Statistical analysis was performed using Statview 5.0.1 software released from Windows Statistical (SAS Institute, Inc., Cary, NC, USA).

Relative quantification was performed via the ΔΔCt method using Bio-Rad’s CFX96 Manager software 3.1.1621 (CFX-96 Real-Time PCR detection systems, Bio-Rad Laboratories Inc., Hercules, CA, USA). Skewed variables were log transformed before statistical analysis. Differences between more than two independent groups were analyzed using Fisher’s test after ANOVA and relations between the variables were assessed by bivariate and simple and multiple linear regression analyses. Results are expressed as mean ± S.E.M. and a *p*-value < 0.05 was considered significant.

## 4. Results

**Figure 1:**
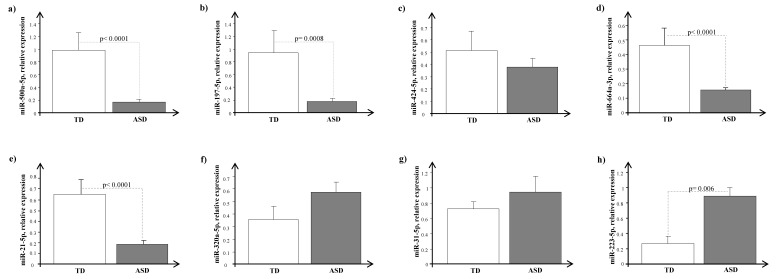
Expression levels of circulating miRNAs, (**a**) miR-500a-5p, (**b**) miR-197-5p, (**c**) miR-424-5p, (**d**) miR-664a-3p, (**e**) miR-21-5p, (**f**) miR-320a-5p, (**g**) miR-31-5p, and (**h**) miR-223-5p, in typically developing (TD, white bar) and autism spectrum disorder (ASD, dark grey bar) children.

At baseline, before probiotic supplementation, we observed different expression patterns of miRNAs between ASD pre-schoolers and TD peers. Specifically, the expression levels of miRNAs involved in ASD (miR-500a-5p, miR-197-5p, miR-424-5p, and miR-664-5p) were markedly reduced in ASD pre-schoolers as compared with TD peers (Figure 1a–d), reaching statistical significance for all except for miR-424-5p.

On the other hand, for miRNAs involved in gastrointestinal disorders, the expression levels of miR-320-5p, miR-31-5p, and miR-223-5p were (*p* = 0.006) higher in ASD than in TD subjects, whereas the levels of miR-21-5p were significantly reduced (*p* < 0.0001) in ASD vs. TD children (Figure 1e–h). As reported in Table 3, we also observed significant positive correlations among the analyzed miRNAs.

**Figure 2:**
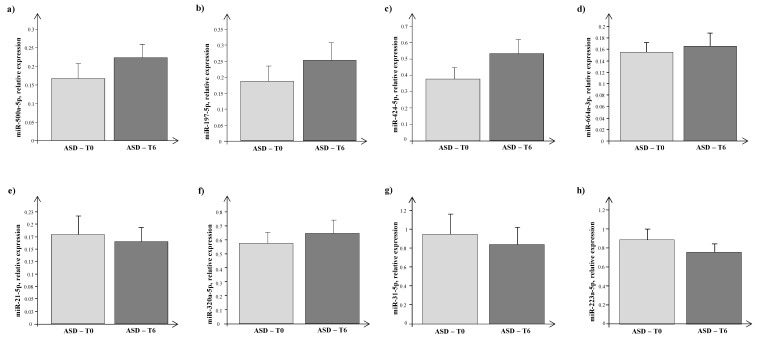
Expression levels of circulating miRNAs, (**a**) miR-500a-5p, (**b**) miR-197-5p, (**c**) miR-424-5p, (**d**) miR-664a-3p, (**e**) miR-21-5p, (**f**) miR-320a-5p, (**g**) miR-31-5p, and (**h**) miR-223-5p, in autism spectrum disorder (ASD) children before (T0, grey bar) and after 6 months of probiotic administration (T1) (dark grey bar).

As shown in Figure 2, a 6-month supplementation with probiotics did not significantly change the expression levels of miRNAs with respect to baseline (T0 vs. T1), considering the ASD population both as a whole (Figure 2a–h) and split into GI and NGI subgroups.

**Figure 3:**
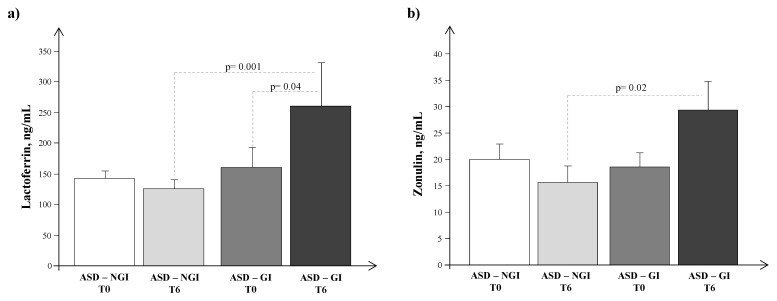
Plasma levels of (**a**) lactoferrin and (**b**) zonulin in autism spectrum disorder (ASD) children split into gastrointestinal (GI) and non-GI (NGI) groups before (T0) and after 6 months of probiotic administration (T1).

Plasma lactoferrin and zonulin levels did not differ in ASD children between T0 and T1 (146.7 ± 13.1 vs. 163.71 ± 23.67 ng/m; *p* = ns and 19.5 ± 2.3 vs. 19.4 ± 2.8 ng/mL; *p* = ns). Circulating lactoferrin levels were higher in the GI group than in the NGI group after administration of probiotics, *p* = 0.04 (Figure 3a,b).

**Figure 4:**
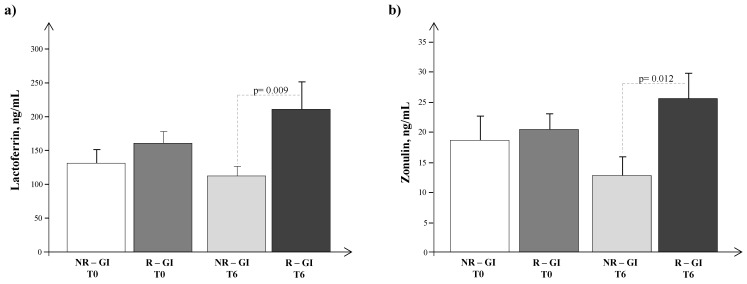
Plasma levels of (**a**) lactoferrin and (**b**) zonulin in the autism spectrum disorder population subdivided into responders (R) and non-responders (NR) as regards gastrointestinal (R-GI/NR-GI) symptoms.

We also compared the plasma levels of lactoferrin and zonulin in R-GI/NR-GI at T0 and T1 (Figure 4a,b), observing slight, non-significantly higher levels of both biomarkers in R-GI at T0 that increased significantly only in R-GI at T1. For R-ASD/NR-ASD, circulating levels of lactoferrin and zonulin were similar at T0 (143.6 ± 13.9 vs 149.9 ± 23.2 ng/mL, *p* = ns; 17.6 ± 2.6 vs. 21.5 ± 3.8 ng/mL, *p* = ns) and T1 (123.8 ± 14.2 vs. 206.4 ± 44.5 ng/mL, *p* = ns; 18.1 ± 3.7 vs. 20.8 ± 4.3 ng/mL, *p* = ns). The expression of miRNAs at T0 and T1 did not differ in R-GI/NR-GI nor in R-ASD/NR-ASD.

**Figure 5:**
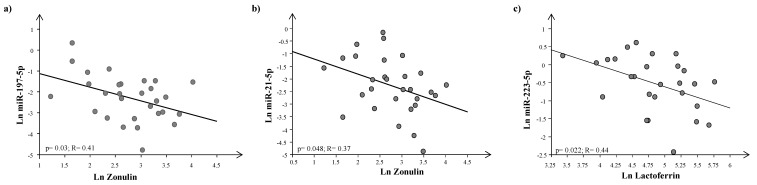
Regression analysis between zonulin plasma levels and (**a**) miR-127-5p and (**b**) miR-21-5p and (**c**) between lactoferrin and miR-223-5p after logarithmic transformation.

We found a significant negative correlation between zonulin and miR-197-5p and miR-21-5p at T0, as well as between lactoferrin and miR-223-5p at T1 (Figure 5).

## 5. Discussion

A crosstalk between mRNAs and miRNAs indicates the existence of a network that plays a critical role in brain development [50], and the putative involvement of these transcripts is reported in the current literature. Dysregulated miRNAs are related to several neurological and neurodevelopmental disorders [51,52,53,54] due to their role in brain function and, in particular, in neuronal plasticity and development [55]. Studies on ASD pathogenesis have shown the possible involvement of miRNAs in ASD development (for a recent systematic review and meta-analysis, see [56]); these molecules, in fact, have been implicated in different cellular processes, such as development, proliferation, differentiation, growth control, homeostasis, and apoptosis. Animal models have shown that synaptogenesis is influenced by miRNAs controlling the expression of genes coding for proteins such as neuroligin and neurexin, which are involved in neurotransmitter release or in synaptic targeting and strongly associated with ASD [57,58,59].

In humans, miRNA measurements have been obtained from different samples, including lymphoblastoid cells [60], the post-mortem cerebral cortex [61], saliva [62], and serum, and possibly have different signatures on the basis of the samples analyzed.

Among the investigations evaluating miRNA in serum samples, [63] thirteen miRNAs were differentially expressed between children and adolescents with ASD (n = 55, age range: 6–16 years) and sex and age matched TD controls (n = 55), some being downregulated (miR-151a-3p, miR-181b-5p, miR-320a, miR-328, miR-433, miR-489, miR-572, and miR-663a) and others being upregulated (miR-101-3p, miR-106b-5p, miR-130a-3p, miR-195-5p, and miR-19b-3p). Crucially, a recent study identified miR-140-3p as differentially upregulated in children with ASD compared not only with TD controls but also with subjects with Tourette’s syndrome (TS) or with TS plus ASD, suggesting its potential role in supporting the differential diagnosis of ASD [64]. Yu and colleagues [65] highlighted that miR-483-3p is upregulated in ASD children, leading to a cascading impact on dendritic and synaptic development, which, in turn, possibly contributed to the pathogenesis of ASD in terms of impairments in intellective functioning and behavior. Upregulated miR-15b-5p was found in both the discovery and replication cohorts of adults with ASD [66]; however, this was in contrast to a previous investigation reporting downregulated expression of peripheral miR-15b-5p in ASD subjects [67].

The heterogeneity of miRNA, evaluated after combining the different analysis methods used, makes the results difficult to compare and still far from having clinical implications and being able to be used as biomarkers for ASD.

In addition to miRNA profiling, multi “omic” profiling methods have been suggested for improving diagnosis accuracy, expanding their application in clinical settings [68].

In this study, we evaluated the transcriptional profile of plasma miR-500a-5p, miR-197-5p, miR-424-5p, and miR-664-5p levels, since these miRNAs have been validated as good candidates for non-invasive molecular biomarkers in ASD patients. The highest diagnostic potential was manifested by miR-500a-5p and miR-197-5p, whose combined ROC curve demonstrated very strong predictive accuracy [17]. When miRNA plasma samples from ASD pre-schoolers were compared with TD children, significantly lower expression levels in ASD subjects were reported [17,63].

The apparent involvement of miR-424-5p in the control of cell division in different tissues could also play a role in ASD pathogenesis. Recently, Wu et al. [69] hypothesized that the evolutionary role of some miRNAs in the primate brain is related to the inhibition of excessive cell proliferation, a phenomenon observed in children with ASD [63,69,70,71,72]. Therefore, the downregulation of miR-424-5p in ASD probands may reflect an insufficient capability to limit early postnatal brain overgrowth.

Additionally, miRNA-500a-5p has been observed to take part in biological processes in the CNS: its expression has been demonstrated in the brain, in the spinal cord, and, with a peculiar asymmetric pattern of expression, in embryonic structures associated with limb development in a murine model during embryogenesis [73].

In addition, since children with ASD frequently have a high prevalence of GI disorders, we also investigated the expression levels of miRNAs such as miR-21-5p, miR-320-5p, miR-31-5p, and miR-223-5p, which are known to be involved in gastrointestinal disorders [18]. Although the expression levels of miR-320-5p, miR-31-5p, and miR-223-5p were significantly higher in ASD than in TD children, the expression of miR-21-5p was significantly lower in ASD children than in their TD peers.

Interestingly, it was recently reported that the expression level of miR-21-5p was upregulated in the brains of ASD patients, suggesting a possible involvement in ASD as well as in gastrointestinal disorders [74].

We also estimated the miRNA expression trend after supplementation with probiotics in ASD children but did not find any significant modifications. It is known that host–microbiota interactions play a vital role in intestinal homeostasis and miRNAs have been considered key molecular regulators mediating such mutualistic relationships. In contrast to our results, some investigations reported that administering probiotics can modify these interactions, influencing the expression of miRNAs [75,76]. We do not have a clear explanation for these different results. However, we cannot exclude that differences in the enrolled populations, in their clinical characteristics, or in the dosage and composition of administered probiotics might have been responsible for the differing results. Interestingly, we found a significant increase in lactoferrin levels in the GI subgroup after probiotic supplementation, which could suggest an improvement in the intestinal barrier function as a consequence of probiotic administration. Lactoferrin has been reported to perform diverse biological functions, including antibacterial activity, anti-inflammatory activity, intestinal barrier protection, and immune cell modulation, and is involved in maintaining intestine mucosal immune homeostasis [77].

The increase in lactoferrin after probiotic supplementation seems to involve the upregulation of miRNA 223: indeed, we detected an inverse relationship between lactoferrin levels and miRNA 223 expression at T1 (Figure 5). Specifically, miRNA 223 acts as pro-inflammatory marker directly targeting Claudin-8, a critical family member in the maintenance of normal intestinal barrier properties [78]. This improvement is also exhibited by an increase in the production of lactoferrin by virtue of its anti-inflammatory activities. Lactoferrin is a first-line defense protein for protection against microbial infections and subsequent development of systemic disease [79,80]. The clinical importance of lactoferrin to control these processes has been clearly demonstrated through a ground-breaking study on neonates [81], where dietary supplementation with lactoferrin reduced the occurrence of late-onset sepsis. Lactoferrin has indeed been proven as a major innate immune responder that is important in the control of the development of acute septic inflammation [82].

High levels of serum zonulin and impaired function of the intestinal barrier have been described in children with neuropsychiatric disorders, including ASD [83]; in the current study, we found an inverse correlation between zonulin and miRna 21 levels at T0 (Figure 5). The finding of increased zonulin levels in the GI responder group after the administration of probiotics is counterintuitive and not straightforward to interpret. We could hypothesize that the improvement of gastrointestinal symptoms observed in the responder group does not directly involve zonulin-mediated mechanisms. Moreover, two recent investigations have detected a direct correlation between zonulin levels and the severity of ASD symptoms evaluated through the Childhood Autism Rating Scale (CARS) [84,85]. In our work, we observed an inverse relationship between the zonulin levels and miRNA 197, a miRNA previously detected as deregulated in ASD [65,86,87].

However, despite compelling evidence for the involvement of miRNAs in neurodevelopment, their contribution to the pathogenesis of ASD has, to date, been inadequately assessed. A few miRNAs have been identified as consistently dysregulated in ASD by independent studies, specifically, miR-144-3p, miR-23b, miR-106b, 150-5p, 320a, 92a-2-5p, 486-3p, and miR-451a [56]. The latter is the only miRNA that was associated with the impairment of social interaction in two independent investigations [88,89]. A systematic characterization of the miRNAs targeting high-confidence ASD genes is likely to provide new insights into the mechanisms underlying ASDs, which in turn may pave the way for designing appropriate miRNA therapeutics for ASDs.

## 6. Limitations

Among the limitations of the current study, we recognize the unequal sample size between ASD patients and TD controls (31 versus 10). The small sample size of the control group is due to the limited availability of TD pre-schoolers who have a venipuncture performed for clinical reasons. Therefore, the current investigation, although giving interesting information, indicates that further studies are needed with larger sample sizes for both the ASD and TD subjects.

Another possible limitation concerns the measurement of miRNAs in the blood. Indeed, a recent systematic review and meta-analysis indicated saliva as the most advantageous biofluid in ASD in terms of detected circulating miRNAs [56]. However, serum miRNAs are known to be remarkably stable, reproducible, and resistant to the actions of RNase, underlying their potential as non-invasive biomarkers for ASD; additionally, the same direction of regulation has been observed in the brain [90].

Furthermore, even if the literature on ASD children is still emerging, we measured lactoferrin and zonulin in the blood as markers of intestinal function. Recent studies confirmed serum zonulin as a potential marker for intestinal permeability [83,91]. Zonulin secretion is modulated by various factors, including nutrition and the microbial composition of the intestinal microbiota; for this reason, the recent literature considers zonulin as a potential therapeutic target in microbiota–gut–brain axis disorders [92]. In addition, we studied lactoferrin because of its antibacterial activity, anti-inflammatory activity, intestinal barrier protection, and immune cell modulation, which makes it a pivotal factor in the maintenance of immune homeostasis of the intestinal mucosa [77]. Therefore, the evaluation of the plasma lactoferrin and zonulin levels in groups of ASD children and their correlations with other clinical and biochemical parameters will provide a further, promising pathway to take into consideration in the study of the gut–brain axis in ASD.

## Figures and Tables

**Table 1:** Demography, clinical characteristics, and body compositions of study populations.

	ASD Subjects (n = 31)	TD Subjects (n = 10)	*p*-Value
Males (%)	77.4	77.7	n.s.
AGE (years)	4.28 ± 0.22	5.00 ± 0.61	n.s.
WEIGHT (kg)	17.62 ± 0.67	15.18 ± 1.45	n.s.
HEIGHT (cm)	104.68 ± 1.62	100.85 ± 5.53	n.s.
BMI (kg/m^2^)	15.93 ± 0.35	14.73 ± 0,11	n.s.
BMI Z-SCORE	0.03 ± 0.26	−0.93 ± 0.29	n.s.

ASD: autism spectrum disorder; TD: typical development; n.s.: not significant; BMI: Body Mass Index.

**Table 2:** Mature miRNA sequence.

Gene	Forward Primer Sequence (5′-3′)	Genbank Accession Number	Location	Ta, °C
**hsa-miR-197-5p**	CGGGTAGAGAGGGCAGTGGGAGG	NR_029583.1	chr 1p13.3	55
**hsa-miR-424-5p**	CAGCAGCAATTCATGTTTTGAA	NR_029946.1	chr Xq26.3	55
**hsa-miR-664a-3p**	TATTCATTTATCCCCAGCCTACA	NR_031705.1	chr 1q41	55
**hsa-miR-500a-5p**	TAATCCTTGCTACCTGGGTGAGA	NR_030224.1	chr Xp11.23	55
**hsa-miR-21-5p**	TAGCTTATCAGACTGATGTTGA	NR_029493.1	chr 17q23.1	55
**hsa-miR-320a-5p**	GCCTTCTCTTCCCGGTTCTTCC	NR_029714.2	chr 8p21.3	55
**hsa-miR-31-5p**	AGGCAAGATGCTGGCATAGCT	NR_029505.1	chr 9p21.3	55
**hsa-miR-223-5p**	CGTGTATTTGACAAGCTGAGTT	LM608368	chr Xq12	55

**Table legend. hsa-miR-197-5p:** homo sapiens microRNA-197 with 5p strand present in the forward position; **hsa-miR-424-5p:** homo sapiens microRNA-424 with 5p strand present in the forward position; **hsa-miR-664-3p:** homo sapiens microRNA-142 with 3p strand present in the reverse position; **hsa-miR-500a-5p**: homo sapiens microRNA-500a with 5p strand present in the forward position; **hsa-miR-21-5p:** homo sapiens microRNA-21 with 5p strand present in the forward position; **hsa-miR-320a-5p:** homo sapiens microRNA-320a with 5p strand present in the forward position; **hsa-miR-31-5p:** homo sapiens microRNA-31 with 5p strand present in the forward position; **hsa-miR-223-5p:** homo sapiens microRNA-31 with 5p strand present in the forward position.

**Table 3:** Significant positive correlations among the analyzed miRNAs.

	miR-197-5p	miR-21-5p	miR-320a-5p	miR-424-5p	miR-500a-5p	miR-664a-5p
**miR-197-5p**	-	R = 0.36; *p* = 0.003	R = 0.35; *p* = 0.004	R = 0.33; *p* = 0.007	R = 0.60; *p* < 0.0001	R = 0.55; *p* < 0.0001
**miR-21-5p**	R= 0.36; *p* = 0.003	-	-	R= 0.29; *p* = 0.02	R = 0.74; *p* < 0.0001	R = 0.64; *p* < 0.0001
**miR-320a-5p**	R= 0.35; *p* = 0.004	-	-	R= 0.45; *p* = 0.002	-	-
**miR-500a-5p**	R= 0.60; *p* < 0.0001	R = 0.74; *p* < 0.0001	-	R = 0.25; *p* = 0.05	-	R = 0.71; *p* < 0.0001

## Data Availability

Data are contained within the article.
